# Electroencephalogram Profiles for Emotion Identification over the Brain Regions Using Spectral, Entropy and Temporal Biomarkers

**DOI:** 10.3390/s20010059

**Published:** 2019-12-20

**Authors:** Noor Kamal Al-Qazzaz, Mohannad K. Sabir, Sawal Hamid Bin Mohd Ali, Siti Anom Ahmad, Karl Grammer

**Affiliations:** 1 Department of Biomedical Engineering, Al-Khwarizmi College of Engineering, University of Baghdad, Baghdad 47146, Iraq; dr.mohannad@uob.edu.iq; 2 Department of Electrical, Electronic & Systems Engineering, Faculty of Engineering & Built Environment, Universiti Kebangsaan Malaysia, UKM Bangi, Selangor 43600, Malaysia; sawal@ukm.edu.my; 3 Department of Electrical and Electronic Engineering, Faculty of Engineering, Universiti Putra Malaysia, UPM Serdang, Selangor 43400, Malaysia; sanom@upm.edu.my; 4 Malaysian Research Institute of Ageing (MyAgeing), Universiti Putra Malaysia, Serdang, Selangor 43400, Malaysia; 5 Department of Evolutionary Anthropology, University of Vienna, Althan strasse 14, A-1090 Vienna, Austria; karl.grammer@univie.ac.at

**Keywords:** emotion, electroencephalography, spectral power, entropy, Hilbert transform, ANOVA, Pearson’s correlation

## Abstract

Identifying emotions has become essential for comprehending varied human behavior during our daily lives. The electroencephalogram (EEG) has been adopted for eliciting information in terms of waveform distribution over the scalp. The rationale behind this work is twofold. First, it aims to propose spectral, entropy and temporal biomarkers for emotion identification. Second, it aims to integrate the spectral, entropy and temporal biomarkers as a means of developing spectro-spatial (SS), entropy-spatial (ES) and temporo-spatial (TS) emotional profiles over the brain regions. The EEGs of 40 healthy volunteer students from the University of Vienna were recorded while they viewed seven brief emotional video clips. Features using spectral analysis, entropy method and temporal feature were computed. Three stages of two-way analysis of variance (ANOVA) were undertaken so as to identify the emotional biomarkers and Pearson’s correlations were employed to determine the optimal explanatory profiles for emotional detection. The results evidence that the combination of applied spectral, entropy and temporal sets of features may provide and convey reliable biomarkers for identifying SS, ES and TS profiles relating to different emotional states over the brain areas. EEG biomarkers and profiles enable more comprehensive insights into various human behavior effects as an intervention on the brain.

## 1. Introduction

Within the brain, impetus inclinations, behavioral reactions, physiological stimulation, states of mind and cognitive procedures are all directly conveyed through emotion. Brain activity and neural pathways are interrelated in a manner that influences mathematical, verbal, perceptive and other forms of intelligence, which further shape emotions [1,2]. From a particular response of the body to an instinctive reaction, individual emotional reactions can vary [3]. Accordingly, the possible extent of congruence between socio-affective circumstances and particular brain areas has been investigated through applying an array of simulation methods in a substantial number of studies [4,5].

The dimensional and discrete models are the two affective models that may be adopted to determine and categorize affective states in accordance with a psychological perspective. Key emotions are identified in the discrete model which specific, distinct affective states are connected to; fundamental emotions such as happiness, sadness, surprise, disgust, fear and anger are individually or in some mixture deemed to be responsible for any further emotions [6,7]. Adopting a circumplex emotion model, the dimensional model has been pervasively adopted for affective identification application mapping, as a two-dimensional (2D) cognitive-emotional state theory [8,9]. A two-scale valence-arousal graph is used for conveying emotions, with emotional strength between calm to excited presented on the vertical axis as ‘arousal’, while the unpleasant to pleasant degree of emotion is conveyed on the horizontal axis as ‘valence’ [8,9,10]. Furthermore, quartiles Q1 to Q4 are the four principal sites of emotional states, with low arousal–high valence (LAHV), low arousal–low valence (LALV), high arousal–low valence (HALV) and high arousal–high valence (HAHV) presented in Q4, Q3, Q2 and Q1 respectively [11]. Beyond the 2D model, focus-disinterest characteristics have been incorporated into a three-dimensional (3D) cognitive-emotional state model in certain studies [12]. 

Various emotional states have been produced through the adoption of varied techniques in studies. Consequently, audio-visual, auditory or visual stimuli have all been adopted in different instances. As in brain-computer interfacing (BCI) research, diminishing or expanding the sensorimotor rhythm amplitude is the process for auditory and visual stimuli [13]. Additionally, compared to music-based audio stimuli, brain signals more straightforwardly convey picture-based visual stimuli [14]. So as to provoke a particular affective state in the most effective manner, auditory and visual stimulus’ amalgamated impact has been acknowledged in studies, thus establishing the optimal context for affective identification. Regarding automatic emotion identification, audio-visual stimuli have also been applied [4]. In contrast with alternative stimuli methods, audio-visual production of emotional states has been found to be superior and more pervasively adopted [13,15,16]. Therefore, brief audio-visual film excerpts were adopted to elicit emotion in this research.

Emotional changes would be elicited using different physiological signals such as galvanic skin response (GSR) [15], electrodermal activity (EDA) [17], blood volume pressure (BVP) [18], and skin temperature (ST) [19], evoked potentials (EP) [20], electrocardiogram (ECG) [21], electromyogram (EMG) [22], and electroencephalogram (EEG) [23,24,25,26,27,28,29,30]. Clinically, EEG signals have been widely used as useful indicators of different mental states such as epilepsy, Alzheimer’s disease (AD) and vascular dementia (VaD) [31,32,33,34,35].

As the brain is a complex structure that has a dynamic behavior, electrical activities including emotional states, can be reflected by using EEG. EEG is a neurophysiological tool used to monitor and identify brain changes [36]. EEG is a widely available, cost-effective, and non-invasive tool that tracks information processing with milliseconds precision and high temporal resolution [36,37]. A typical clinical EEG frequency ranges from 0.01 Hz to approximately 70 Hz [38]; the corresponding waveforms have an amplitude of a few µVolt to approximately 100 µVolt [39]. EEG background waveforms also convey valuable information. Thus, these waveforms can be classified into five specific frequency power bands: the delta band (δ), the theta band (θ), the alpha band (α), the beta band (β), and the gamma band (γ) [40,41]. Studies on EEG signal processing have been conducted to identify the brain activity patterns involved in cognitive science, neuropsychological research, clinical assessments, and consciousness research [42,43,44,45,46,47]. Recently, EEG has been widely used to assess and evaluate the human emotional states with excellent time resolution [3,15,28,29,30,48]. EEG can provide useful information of emotional states that have been described as a potential biomarker to evaluate different emotional responses from multi-channel EEG datasets over the brain regions [38]. A key advantage of the multi-channel EEG signal processing is to interpret EEG changes during different emotional states over the brain regions. Thus, numerous studies have been performed including this study to deal with issue [49,50,51]. For instance, Nattapong et al. have proposed a continuous music-emotion recognition approach based on brain wave signals [52]. Olga et al. have applied a combined music therapy process with the real-time EEG-based human emotion recognition algorithm to identify the current emotional state based on neurofeedback and adjust the music therapy based on the patient’s needs [53]. 

As the brain neurons are controlled by linear and non-linear phenomena, several linear techniques including traditional spectral powers have been used to analyze the smoothness of the EEG as a time series and to elicit the emotional information from the EEG signals. The higher order statistics (HOS) features, namely skewness and kurtosis, have been applied as well to measure the presence of transients in the signal [52,54,55,56]. Due to the capability of the brain to perform sophisticated emotional tasks and to investigate the complex dynamic information that is reflected from the brain cortex, several researchers have used non-linear methods for automatic detection of emotions through EEG signals [57]. Previous emotion studies have used a small number of features, mostly relative powers [3], Hurst [15], Hjorth parameters [58], Fractal Dimension (FD) [59], and statistical features [60,61]. Moreover, entropy has been considered as the most prevalent methods to evaluate the presence or absence of long-range dependence on physiological signal analysis including approximate entropy (ApEn), sample entropy (SampEn) and permutation entropy (PerEn) which are relatively robust to noise and powerful enough to quantify the complexity of a time series [62]. Amplitude-aware permutation entropy (AAPE) has demonstrated efficiency in discriminating between calmness and distress [63,64,65]. Fuzzy entropy (FuzEn) was proposed for EEG analysis in [66,67]. SampEn is slightly faster than FuzEn, however the latter is more consistent and less dependent on the data length [68]. Azami et al. has considered the advantages of FuzEn over SampEn and recently has introduced refined composite multiscale FuzEn (RCMFE) [68,69,70]. In RCMFE, the entropy stability is improved, the signals’ length sensitivity is reduced and the coarse-grained process of RCMFE smoothens the signals. Hence, RCMFE has been used in this study. 

EEG signal contains useful information on physiological states of the brain and has proven to be a potential biomarker to realize the linear and non-linear behavior of the brain [31,32,71,72,73]. Therefore, motivation of this work is twofold. First, in order to investigate alternate information from multi-channel emotional EEG datasets, linear spectral conventional analysis, non-linear entropy method and temporal feature were performed to obtain the potential EEG emotional biomarkers. Second, the obtained biomarkers may be further considered to provide additional information to illustrate the EEG spectro-spatial (SS), entropy-spatial (ES) and temporo-spatial (TS) profiles for the seven emotional states over the brain regions.

The preprocessing stage has been used to limit the unwanted frequency from the EEG dataset. Spectral biomarkers were computed by employing the absolute powers (AbsP) of δ, θ, α, β, and γ. Moreover, to quantify the complexity of brain functions, entropy biomarkers across multi-channel EEG signals have been measured using RCMFE. Furthermore, the temporal biomarker was reported by amplitude envelope which was extracted using Hilbert transform, and the amplitude values were investigated by skewness (Skw) to get $H_{Skw}$. Three stages of two-way analysis of variance (ANOVA) were conducted to obtain the spectral, entropy and temporal biomarkers followed by Pearson’s correlations to get the spatial profiles that are related to anger, anxiety, disgust, happiness, sadness, surprise and neutral emotional states over the brain regions. The valance-arousal circumplex model was employed in this study to represent and recognize human emotions due to its effectiveness in viewing the emotions while audio-visual video clips were used [74]. To the author’s best knowledge this study has two contributions: firstly, it is the first use of a combination of certain features to develop spectral, entropy and temporal biomarkers towards SS, ES and TS profile identification for the seven emotional states over the brain regions; secondly, the EEG elicitation protocol and EEG measurement procedure have never been used before for emotion data acquisition.

## 2. Materials and Methods

**Figure 1:**
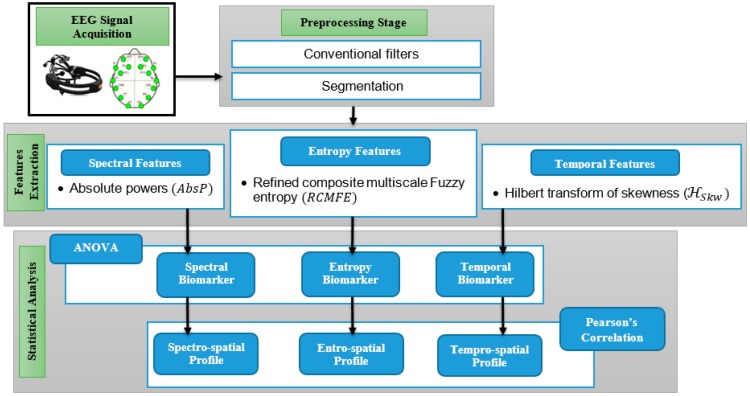
The block diagram of the proposed study.

This study is intended to be focused on the potential EEG emotional biomarkers and profiles that obtained from EEG datasets. Figure 1 illustrates the block diagram of the proposed study.

### 2.1. EEG Acquisition and Recording

A transportable Emotiv EPOC EEG 14-channel headset (Emotive Systems, Inc., San Francisco, CA, USA) was adopted in order to evaluate 14 EEG electrodes (AF3, F7, F3, FC5, T7, P7, O1, O2, P8, T8, FC6, F4, F8, AF4) overall with 2 ground electrodes which were provided by the driven right leg (DRL) mastoid and common mode sense (CMS) left mastoid. The Emotiv EPOC EEG uses sponge-based electrodes which were located based on the 10–20 system. The electrode information was filtered through a 0.5–70 Hz band-pass filter. A 128 Hz sampling frequency was used with a resolution of 0.51 mV. 

**Figure 2:**
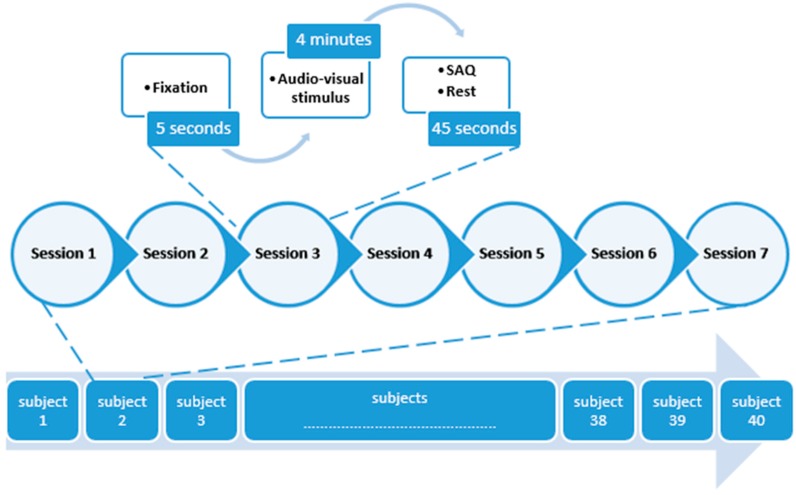
The experimental protocol of emotion.

40 university students agreed to participate in this research (Table 1). A subject appraisal was carried out for each individual to guarantee that no previous psychiatric or neurological problems had been suffered, with the participant then providing their agreement to participation through signing an informed consent document, before the study proceeded. Subjects were presented with different brief film excerpts alongside audio that aimed to be emotionally engaging, after which a self-assessment questionnaire (SAQ) was filled in by the subjects to provide their assessment and scoring of emotional reactions to the excerpts. The subsequent video excerpt was presented after a 45 s pause. This process is presented in Figure 2 [75].

Respondents rated their responses in the SAQ according to the level of emotion felt, from 5 = very high; 4 = high; 3 = medium; 2 = low, to 1 = very low, thus providing a five-point scale [48]. This enabled the neutral circumstances and six affective states—anger, anxiety, sadness, disgust, surprise and happiness. Rottenberg’s suggestions were followed in order to identify appropriate affective film excerpts [75]; one film excerpt had a duration of four minutes, which was the longest, with the others differing in length. 

In order to assist with the presentation of the affective video excerpts to the subjects, the University of Vienna’s virtual emotion presenter program was applied. Further information source documentation and arbitrary presentation is permitted through the program. The anthropology research laboratory was the location of the research; the sound for the film was played to the subjects at a consistent and reasonable volume through a stereo system; film excerpts were viewed on an LCD display; the laboratory had consistent natural light sources, with the VEP also adopted as explained above. As the 40 subjects viewed the affective video excerpts, monitoring of the EEG electrode signals ensued. 

### 2.2. Preprocessing Stage

Brain responses and artefacts may have intersected due to the latter residing within the frequency bands of the EEG waves. In terms of EEG signal preprocessing, a significant aspect is noise eradication. Standard filtering may be an aspect of the preprocessing phase, with EEG signals seeing the introduction of further software filters—band pass and notch filters for example—to carry out this process. As a means of restricting EEG signal frequencies in accordance with [76], a higher cutoff frequency around 64 Hz and a low cutoff of 0.5 Hz (3 dB) was adopted for the band pass filter. A 50 Hz cutoff frequency was adopted for the notch filter; eliminating A/C electricity line interference is the typical reason for doing so [38]. Three 10 s trials per video excerpt comprised the overall video, with every 10 s trial comprising of 1280 information points, in order to carry out additional filtered EEG data processing.

### 2.3. Features Extraction

Comprehending various affective states’ associations is assisted by EEG signals as a significant source of brain function data. In terms of identifying particular emotional actions, the EEG signal offers various quantifiable measures. Accordingly, affective EEG biomarkers were derived from a number of characteristics, primarily distinguished into temporal, entropy and spectral power characteristics, as a means of determining the principal characteristics that enable the EEG data to be matched with affective states, while also allowing improved explication of the brain areas’ altering affective states. Additionally, the brain areas’ and seven affective states’ spectro-spatial (SS), entropy-spatial (ES) and temporo-spatial (TS) profiles were determined through combining the quantified biomarkers via Pearson’s correlations. 

#### 2.3.1. Spectral Biomarker

Through investigating the impact on different brain areas of various multi-channel EEG signals’ frequency bands, such spectral assessments as a linear characteristic for appraising affective alterations have been pervasively adopted. Multi-channel EEG alterations have been quantified via the AbsP characteristic as aspects of brain rhythms. Meanwhile, Welch’s technique was applied to calculate the EEG information’s power spectral density (PSD), with the specific frequency bands of gamma (γ: 32 ≤ f ≤ 60) Hz, beta (β: 16 ≤ f ≤ 32) Hz, alpha (α: 8 ≤ f ≤ 16) Hz, theta (θ: 4 ≤ f ≤ 8) Hz, and delta (δ: 0 ≤ f ≤ 4) Hz being distinguished as particular frequency bands for the EEG signals’ PSD [77]. A single band’s level of EEG activity autonomous of different bands’ activity is indicated by the AbsP, with Equation (1) adopted to determine it [78].

(1) $$AbsP\left(\%\right)=\frac{10\times \log_{10}\sum^{\;}\mathrm{Selected}\;\mathrm{frequency}\;\mathrm{band}\;}{\sum^{\;}\mathrm{Total}\;\mathrm{range}\;\left(0.5-64\;\mathrm{Hz}\right)}$$

Every film excerpt’s last 30 s were divided into three 10 s parts comprising of 1280 information points per part, providing 3840 information points overall from which the EEG signal information’s AbsP features were ascertained. 

#### 2.3.2. Entropy Biomarker

The obtained EEG signals, inclusive of the RCMFE, have been assessed by applying the non-linear entropy method, given that complex mental procedures may be undertaken by the brain.

In order to calculate the RCMFE based on mean $\left(RCMFE_{\mu }\right)$ for 1 ≤ u ≤ τ, $z_{u}^{\tau }=\left\{y_{u,1}\left(\tau \right),\;y_{u,2}\left(\tau \right),\;\ldots \right\}$, where

$$\mu_{y_{u,j}}\left(\tau \right)=\frac{\sum_{b=u+\tau \left(j+1\right)}^{u+\tau j-1}x_{b}}{\tau }$$

For a defined scale factor τ and embedding dimension, m, $\emptyset_{\tau ,k^{m}}|\left(k=1,\ldots ,\tau \right)$ and $\emptyset_{\tau ,k^{m+1}}|\left(k=1,\ldots ,\tau \right)$ for each of $z_{k}^{\tau }|\left(k=1,\ldots ,\tau \right)\;$are separately calculated. Next, the average of values of $\emptyset_{\tau ,k^{m}}$ and $\emptyset_{\tau ,k^{m+1}}$ on 1 ≤ k ≤ τ are computed, respectively. Finally, the RCMFE is computed as in Equation (2):

(2) $$RCMFE\left(X,\;\tau ,m,\;n,\;r\right)\;=\;-\ln\left({\bar{\emptyset }}_{\tau }^{m+1}/{\bar{\emptyset }}_{\tau }^{m}\right)$$

The embedding dimension m, RCMFE power n, and tolerance r for all of the approaches were respectively chosen as τ=1, m=3, n=2, r = 0.1~0.2SD, and SD is the standard deviation of the original time series [68].

#### 2.3.3. Temporal Biomarker

In contrast with alternative brain imaging approaches, greater temporal resolution and altered temporal changeability over a particular time period are provided by EEG signals, which provide its clinical advantage. Accordingly, precise millisecond readings of electro-physiological alterations may be derived from EEG. Consequently, temporal data analysis enables the formulation of EEG biomarkers. 

The Hilbert transformation’s adoption enables the application of the amplitude envelope to define the temporal biomarkers. Skewness (Skw) was calculated to get $H_{Skw}$ in relation to the distribution for every EEG channel, once the amplitude envelope had been established.

Therefore, to compute the temporal biomarkers set, first the EEG signal $X_{k}\left(n\right)$, for channel k and n is the time-domain index, the temporal envelope is then extracted using the Hilbert transform H{.} as in Equation (3) [79].

(3) $$e_{i}\left(n\right)=\sqrt{X_{k}{\left(n\right)}^{2}+H{\left\{X_{k}\left(n\right)\right\}}^{2}}$$

Let $m_{n}=E\left\{{\left(x-E\left\{x\right\}\right)}^{n}\right\}$ be the $n^{th}$ central moment of the $H_{Skw}$ distributions. The Skw is defined as in Equation (4).

(4) $$Skw=\frac{m_{3}}{{\left(m_{2}\right)}^{3/2}}$$

Skw is the normalized 3rd order moment of amplitude distribution. If the distribution is symmetrical, then Skw is zero. By contrast, large Skw values are associated with the asymmetry degree of amplitude distribution [80,81,82].

### 2.4. Statistical Analysis 

Enhanced comprehension of brain states is a requisite outcome of the approach taken to the EEG dataset’s mapping. IBM USA’s SPSS program version 25 was adopted to undertake statistical analysis. Resultantly, four recording areas relating to the cerebral cortex’s scalp region formed the basis of the initial categorization of the 40 fit participants’ EEG dataset. The dimension of the feature matrix was (40 subjects × 14 EEG Channels × 7 emotional states) = 3920 attributes for each of spectral, entropy and temporal biomarkers. The different brain regions’ alternative affective states’ profiles and affective biomarkers could be directly conveyed by the divergences in brain areas, facilitated by the mean characteristics of the area. The area mean’s derived characteristics were used to categorize the various brain regions’ differences, for example occipital (O1 and O2 channels), parietal (P7 and P8 channels), temporal (T7 and T8 channels) and frontal (AF3, F7, F3, FC5, F4, FC6, F8, and AF4 channels). Subsequently, Levene’s test for homoscedasticity was applied, and the Kolmogorov–Smirnov test was performed to test the normality assumption required by the ANOVA statistical test. Two methods of statistical analysis were applied. One established the extent to which brain areas had varying affective states in relation to temporal, entropy and spectral characteristics, namely the analysis of variance (ANOVA) test. The brain areas’ various connectivity characteristics were established through Pearson’s correlation.

#### 2.4.1. ANOVA

There were three aspects to the ANOVA test. Firstly, the distinctive characteristics were subject to a two-way ANOVA test; the dependent variable related to the spectral biomarker and was the AbsP characteristic, while the independent variables were the four brain areas (occipital, parietal, temporal and frontal) as well as the seven emotional states (anger, anxiety, disgust, happiness, sadness, surprise and neutral). 

Secondly, the RCMFE characteristic was subjected to the two-way ANOVA test, with the independent variables being the brain areas and seven affective states, while the entropy biomarker was the dependent variable.

Thirdly, the dependent variable of the seven affective states’ amplitude envelope distributions’ $H_{Skw}$ was subject to the two-way ANOVA test, with the temporal biomarker’s independent variable being the seven affective states and four brain areas.

Duncan’s test was applied in order to provide the post hoc contrast, with p ˂ 0.05 established as each statistical assessments’ level of significance. Resultantly, the seven affective states and the brain areas’ possible temporal, entropy and spectral biomarkers are conveyed in this part. The Bonferroni post hoc test has been conducted to examine multiple comparisons for each group of tests, including the seven emotional states and the four brain regions.

#### 2.4.2. Pearson’s Correlations

As a means of analyzing and revealing the spatial variability and distribution changes in three different ways along the EEG signals’ length, the spectral, entropy and temporal biomarkers obtained during the previous section will be integrated into the brain spatial information, thus enabling an appropriate understanding of emotional significance. Consequently, three stages of Pearson’s correlation were implemented for developing spectro-spatial (SS), entropy-spatial (ES) and temporo-spatial (TS) profiles. These patterns offered a concise, consolidated method of EEG profile representation over the brain regions relating to anger, anxiety, disgust, happiness, sadness, surprise and neutral emotions. 

During each of the three sessions, Pearson’s correlation coefficient (r) was calculated so as to establish the biomarkers’ correlations, including for the neutral emotional state and six emotion states. Each correlation analysis under the Pearson’s correlation method was calculated at p < 0.05, reflecting statistical significance. All correlation sessions were implemented for every participant.

Determination of every specific affective state’s SS profile—$\left(SS_{anger}\right)$ for anger, $\left(SS_{anxiety}\right)$ for anxiety, $\left(SS_{disgust}\right)$ for disgust, $\left(SS_{happiness}\right)$ for happiness, $\left(SS_{sadness}\right)$ for sadness, $\left(SS_{surprise}\right)$ for surprise)—as well as $SS_{neutral}$ for the neutral affective state, was undertaken during Pearson’s correlation’s initial application.

A 2nd session of Pearson’s correlation, the ES profile—$\left(ES_{anger}\right)$ for anger, $\left(ES_{anxiety}\right)$ for anxiety, $\left(ES_{disgust}\right)$ for disgust, $\left(ES_{happiness}\right)$ for happiness, $\left(ES_{sadness}\right)$ for sadness, $\left(ES_{surprise}\right)$ for surprise—as well as $ES_{neutral}$ for the neutral affective state, were computed.

A 3rd session of Pearson’s correlation, the TS profile—$\left(TS_{anger}\right)$ for anger, $\left(TS_{anxiety}\right)$ for anxiety, $\left(TS_{disgust}\right)$ for disgust, $\left(TS_{happiness}\right)$ for happiness, $\left(TS_{sadness}\right)$ for sadness, $\left(TS_{surprise}\right)$ for surprise)—as well as $TS_{neutral}$ for the neutral affective state, were obtained. 

## 3. Results

An overall duration of 3840 information points over 30 s intervals for the 14 EEG channels was subject to characterization. For the seven specific affective activities, the EEG recordings were distinguished into 10 s parts with 1280 information points being the duration per section. The statistical analysis methods of ANOVA and Pearson’s correlation were applied to determine the character extraction findings.

### 3.1. ANOVA Results 

The subsequent parts explore the four brain areas (occipital, parietal, temporal and frontal) in relation to the seven affective states (anger, anxiety, disgust, sadness, surprise, happiness and neutral) in relation to the spectral, entropy and temporal biomarker statistical features. 

**Figure 3:**
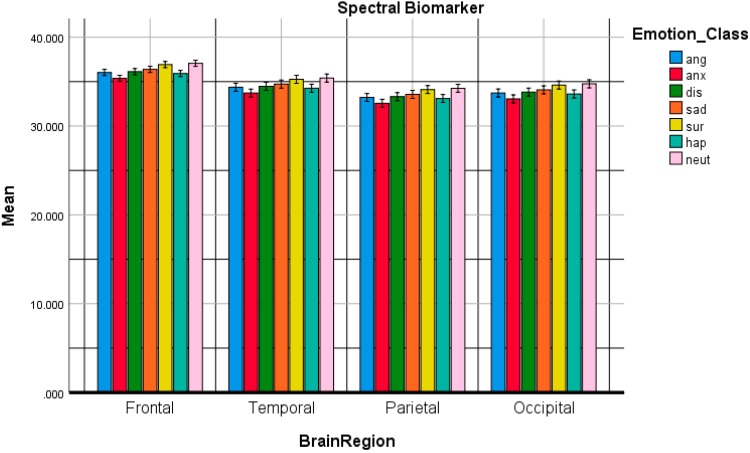
The comparative plot of the spectral biomarker for anger, anxiety, disgust, happiness, sadness, surprise and neutral emotional states over the frontal, temporal, parietal and occipital brain regions.

Figure 3 presents the spectral biomarker performance corresponding to each individual emotional state across the brain regions. It is apparent that the frontal lobes presented the most significant activity compared with other brain lobes $\left(Spectral_{Frontal}>Spectral_{Temporal}>Spectral_{Occipital}>Spectral_{Parietal}\right)$. The highest means were attained for $Spectral_{neutral}$, which varied significantly from all other emotional states across each brain region apart from $Spectral_{surprise}$. Moreover, the $Spectral_{anger}$ response was significantly different from $Spectral_{surprise}\;$and $Spectral_{neutral}$, given that both anger and surprise emotions were situated in the upper-right quadrant and upper-left quadrant of the valance-arousal circumplex model respectively. $Spectral_{anxiety}$ was significantly different from $Spectral_{sadness}$, $Spectral_{surprise}\;$and $Spectral_{neutral}$, with anxiety and sadness located in the upper- and lower-right quadrants of the valance-arousal circumplex model respectively. Meanwhile, surprise was located in the upper-left quadrant of the valance-arousal circumplex model. The significant differences were established at p<0.05 level of significance. 

The Bonferroni post hoc test has been conducted to examine multiple comparisons. Table 2 shows the post-hoc emotion multiple comparisons using Bonferroni adjustments for Spectral Biomarker. The post hoc tests using the Bonferroni correction revealed that neutral was statistically significant from sadness and happiness respectively (p = 0.004, 0.05), anger was statistically significant from sadness and happiness (p = 0.05, 0.05), anxiety was statistically significant from sadness and happiness respectively (p = 0.019, 0.002), sadness was statistically significant from surprise (p = 0.001) and surprise was statistically significant from happiness (p = 0.05). Moreover, from Table 3, the brain region multiple comparisons using Bonferroni adjustments for Spectral Biomarker have been illustrated. The frontal region was statistically significant from temporal, parietal and occipital brain regions (p = 0.05).

**Figure 4:**
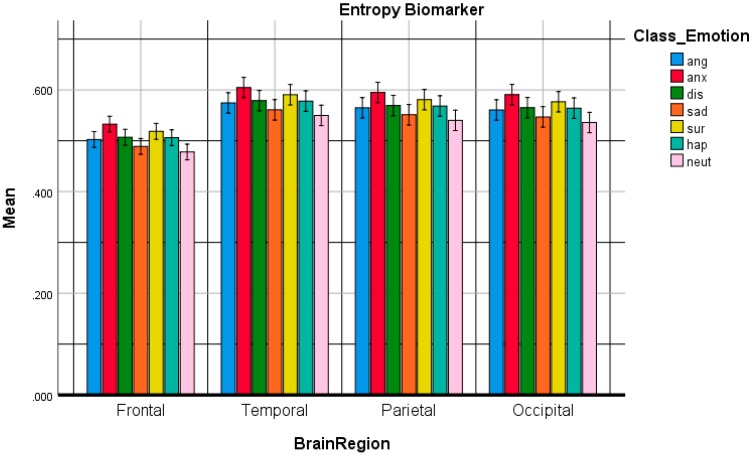
The comparative plot of the entropy biomarker for anger, anxiety, disgust, happiness, sadness, surprise and neutral emotional states over the brain regions.

Secondly, ANOVA was conducted as a comparative study to check the performance of RCMFE entropy biomarkers. The significant differences among the entropy biomarker were evaluated over the four brain regions. The significances were set at p ˂ 0.05. The temporal lobes have the highest mean and they were significantly different from other brain lobes for all emotional states. From the visual inspection of Figure 4, it can be observed that the highest entropy values were noted for the all emotional states $\left(Entropy_{Temporal}>Entropy_{Parietal}>Entropy_{Occipital}>Entropy_{Frontal}\right)$. From the visual inspection of Figure 4, it can be observed that the highest entropy values were noted for the $Entropy_{anxiety}$ has highest mean which was significantly differenced from all emotional states except for $Entropy_{surprise}$. The response of $Entropy_{neutral}$ was significantly different from $Entropy_{anxiety}$ and $Entropy_{surprise}$ as both anxiety and surprise were located at the upper right and upper left quadrant of the valance-arousal circumplex model, respectively. The significant differences were set at ( p<0.05).

For the entropy biomarkers, Table 4 shows the post hoc emotion multiple comparisons using Bonferroni adjustments for the entropy biomarker. The post hoc tests using the Bonferroni correction revealed that anger was statistically significant from disgust and happiness respectively (p = 0.001, 0.05) and sadness was statistically significant from happiness (p =0.0035). Moreover, from Table 5, the brain region multiple comparisons using Bonferroni adjustments for the entropy biomarker have been illustrated. The frontal region was statistically significant from temporal, parietal and occipital brain regions (p =0.05).

**Figure 5:**
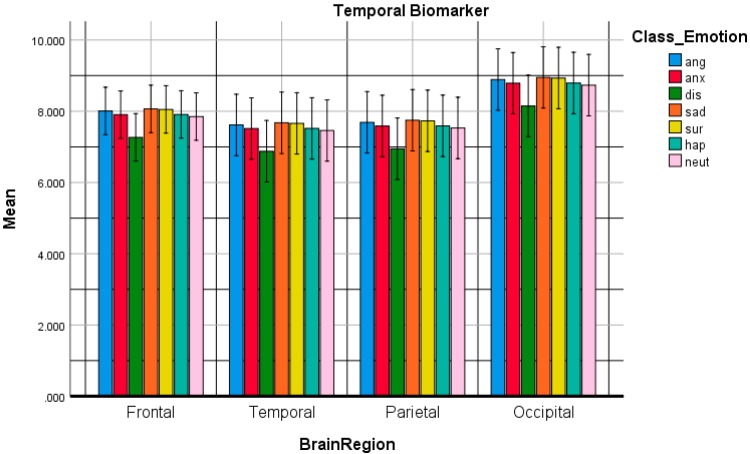
The comparative plot of the temporal biomarkers for anger, anxiety, disgust, happiness, sadness, surprise and neutral emotional states over the brain regions.

Thirdly, ANOVA was conducted as a comparative study to check the performance of temporal biomarkers which have been characterized by the amplitude envelope parameters using $H_{Skw}$ as temporal biomarkers. The significant differences among the temporal biomarkers were evaluated over the 4 brain regions. The significances were set at p ˂ 0.05. Figure 5 shows the temporal biomarkers of the emotional responses among the brain regions. The frontal lobes have the most significant activity in comparison to other brain lobes: $\left(Temporal_{Frontal}>Temporal_{Occipital}>Temporal_{Parietal}>Temporal_{Temporal}\right)$. The response to the $Temporal_{sadness}\;$has the highest mean. $Temporal_{anger}$ and $Temporal_{surprise}$ almost have the same mean, $Temporal_{neutral}$ and $Temporal_{anxiety}$ almost have the same performance and finally $Temporal_{disgust}$ and $Temporal_{happiness}$ have the same effect and that related to their distribution within the valance-arousal circumplex model. The significant differences were set at p<0.05.

For the temporal biomarkers, Table 6 shows the post hoc emotion multiple comparisons using Bonferroni adjustments for the temporal biomarker. The post hoc tests using the Bonferroni correction revealed that it were not statistically different (p >0.05) for the seven emotional states.. Moreover, from Table 7, the brain region multiple comparisons using Bonferroni adjustments for the spectral biomarker have been illustrated. The frontal region was statistically significant from temporal, parietal and occipital brain regions respectively (p =0.05, 0.05, 0.001).

### 3.2. Results of Pearson’s Correlations

During the second statistical analysis stage, Pearson’s correlation coefficients were calculated relating to the spectral, entropy and temporal biomarkers for the neutral state as well as six emotional states (anger, anxiety, disgust, happiness, sadness and surprise) per each EEG channel for the frontal, temporal, parietal and occipital brain regions. Significant differences were calculated as existing between the various emotions with regard to EEG-based correlation alterations. 

**Figure 6:**
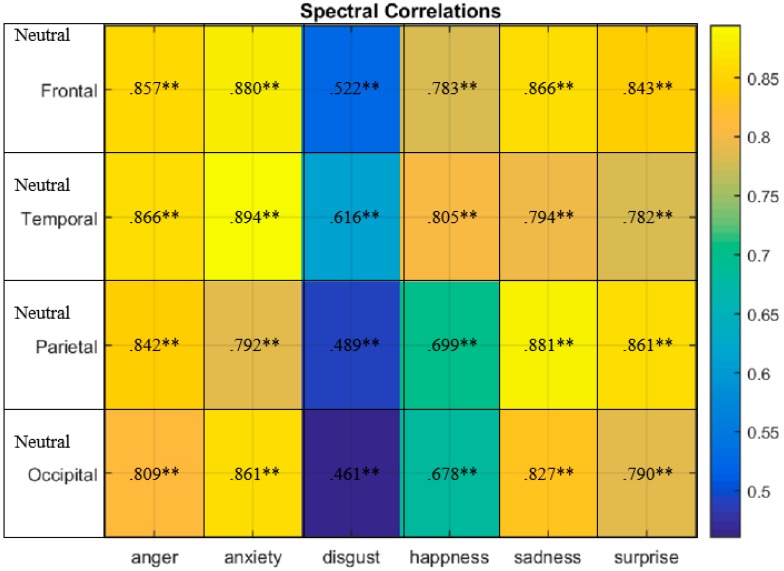
$SS_{neutral}-SS_{anger,\;anxiety,\;disgust,hapiness,sad,surprise}\;$correlations occurring in the frontal, temporal, parietal and occipital brain regions. Correlations of significance at 0.05 level (2-tailed).

The correlations of $SS_{neutral}-SS_{anger,\;anxiety,\;disgust,hapiness,sad,surprise}$ were significantly positive in all cases, as Figure 6 presents. For example, the frontal region $SS_{neutral}$ showed a very strong positive correlation especially with $SS_{anxiety}\;$(r = 0.880, p ˂ 0.01), $SS_{sadness}$ (r = 0.866, p ˂ 0.01) and $SS_{anger}\;$(r = 0.857, p ˂ 0.01). Furthermore, temporal area $SS_{neutral}$ had a very strong positive correlation particularly with $SS_{anxiety}\;$(r = 0.894, p ˂ 0.01), $SS_{anger}$ (r = 0.866, p ˂ 0.01) and $SS_{happiness}$ (r = 0.805, p ˂ 0.01). $SS_{neutral}$ expressed a very strong positive correlation with $SS_{sadness}$, $SS_{surprise}\;$and $SS_{anger}$ (r = 0.881, r = 0.861, r = 0.842, p ˂ 0.01), respectively, in the parietal region. Moreover, $SS_{neutral}$ had a very strong positive correlation with $SS_{anxiety}$, $SS_{sadness}\;$and $SS_{anger}\;(r\;$= 0.861, r = 0.827, r = 0.861, p ˂ 0.01) in the occipital region. Overall, $SS_{neutral}$ and $SS_{anger}$ had the highest correlation in the temporal region (r = 0.866, p ˂ 0.01). $SS_{neutral}$ and $SS_{anxiety}$ had the highest correlation in the temporal region and frontal regions (r = 0.894, r = 0.880, p ˂ 0.01). $SS_{neutral}$ and $SS_{happiness}$ had the highest correlation in the temporal region (r = 0.805, p ˂ 0.01). $SS_{neutral}$ and $SS_{disgust}$ had the highest correlation in the temporal region (r = 0.616, p ˂ 0.01). $SS_{neutral}$ and $SS_{sadness}$ had the highest correlation in the parietal region (r = 0.881, p ˂ 0.01). $SS_{neutral}$ and $SS_{surprise}$ had the highest correlation in the parietal region (r = 0.861, r = 0.970, p ˂ 0.01). Regarding the SS profile, the lowest positive correlation was observed between $SS_{neutral}$ and $SS_{disgust}$ in the occipital and parietal regions (r = 0.461, r = 0.489, p ˂ 0.01) respectively. Accordingly, regarding the SS emotional profile, the frontal, temporal and parietal lobes participated to the greatest extent in emotional elicitation.

**Figure 7:**
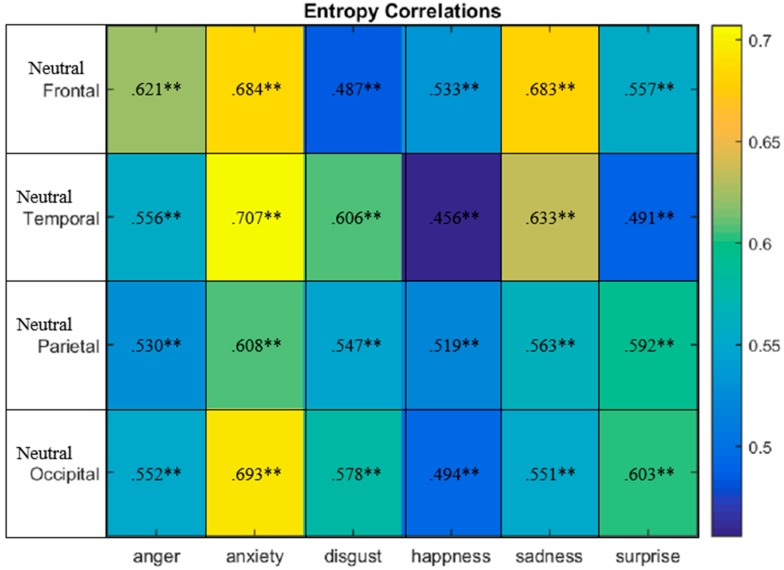
$ES_{neutral}-ES_{anger,\;anxiety,\;disgust,hapiness,sad,surprise}$ correlations occurring in the frontal, temporal, parietal and occipital brain regions. Correlations of significance at 0.05 level (2-tailed).

The correlation of $ES_{neutral}-ES_{anger,\;anxiety,\;disgust,hapiness,sad,surprise}$ had significant positive correlations in all cases, as shown in Figure 7. For instance, in the frontal region, $ES_{neutral}$ had a very strong positive correlation particularly with $ES_{anxiety}\;$(r = 0.684, p ˂ 0.01) and $ES_{sadness}$ (r = 0.683, p ˂ 0.01). It can be observed that for the temporal region that the $ES_{neutral}$ had very strong positive correlation particularly with $ES_{anxiety}\;$(r = 0.707, p ˂ 0.01) and $ES_{sadness}$ (r = 0.633, p ˂ 0.01). For the parietal region, $ES_{neutral}$ had a strong positive correlation particularly with $ES_{anxiety}\;$(r = 0.608, p ˂ 0.01). Moreover, $ES_{neutral}$ had a very strong positive correlation with $ES_{anxiety}\;$(r = 0.693, p ˂ 0.01) at the occipital region. In other words $ES_{neutral}$ and $ES_{anger}$ had the highest correlation at the frontal region (r = 0.621, p ˂ 0.01). $ES_{neutral}$ and $ES_{anxiety}$ had the highest correlation at the temporal region (r = 0.707, p ˂ 0.01). $ES_{neutral}$ and $ES_{disgust}$ had the highest correlation at temporal region (r = 0.606, p ˂ 0.01). $ES_{neutral}$ and $ES_{happiness}$ had the highest correlation at frontal region (r = 0.533, p ˂ 0.01). $ES_{neutral}$ and $ES_{sadness}$ had the highest correlation at the frontal region (r = 0.688, p ˂ 0.01). $ES_{neutral}$ and $ES_{surprise}$ had the highest correlation at the parietal region (r = 0.592, p ˂ 0.01). For the ES profile the lowest positive correlation can be seen between $ES_{neutral}$ and $ES_{happiness}$ at the temporal region (r = 0.456, p ˂ 0.01). Therefore, for the ES emotional profile the frontal lobes were mostly participating in anger, happiness and sadness emotional states, whereas the temporal lobes were responsible for anxiety and disgust emotional elicitation.

**Figure 8:**
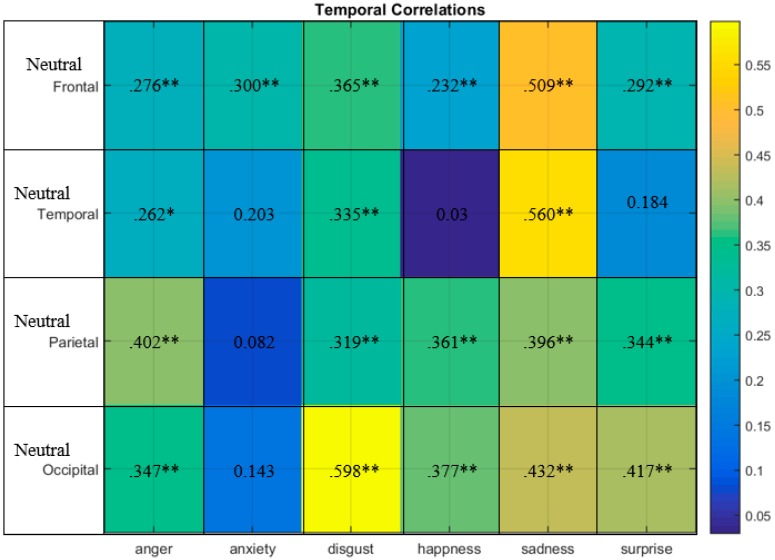
$TS_{neutral}-TS_{anger,\;anxiety,\;disgust,hapiness,sad,surprise}\;$correlations occurring in the frontal, temporal, parietal and occipital brain regions. Correlations of significance at 0.05 level (2-tailed).

The correlation of $TS_{neutral}-TS_{anger,\;anxiety,\;disgust,hapiness,sad,surprise}$ is shown in Figure 8. For instance, in the frontal region, $TS_{neutral}$ had a moderate positive correlation particularly with $TS_{sadness}\;$(r = 0.509, p ˂ 0.01). It can be observed that for the temporal area the $TS_{neutral}$ had a moderate correlation particularly with $TS_{sadness}\;$(r = 0.506, p ˂ 0.01). $TS_{neutral}$ had a moderate positive correlation with $TS_{anger}$ (r = 0.402, p ˂ 0.01) at the parietal region, respectively. Moreover, $TS_{neutral}$ had a moderate positive correlation with $TS_{disgust}\;(r$ = 0.598, p ˂ 0.01) at the occipital region. In other words $TS_{neutral}$ and $TS_{anger}$ had the highest correlation at the parietal region (r = 0.402, p ˂ 0.01). $TS_{neutral}$ and $TS_{anxiety}$ had the highest correlation at the frontal region (r = 0.300, p ˂ 0.01). $TS_{neutral}$ and $TS_{disgust}$ had the highest correlation at the occipital region (r = 0.598, p ˂ 0.01). $TS_{neutral}$ and $TS_{happiness}$ had the highest correlation at the occipital region (r = 0.377, p ˂ 0.01). $TS_{neutral}$ and $TS_{sadness}$ had the highest correlation at the temporal region (r = 0.560, p ˂ 0.01). $TS_{neutral}$ and $TS_{surprise}$ had the highest correlation at the occipital region (r = 0.417, p ˂ 0.01). For the TS profile the lowest positive correlation was observed between $TS_{neutral}$ and $TS_{happiness}$ at the temporal region (r = 0.03, p ˂ 0.01). Therefore, for the TS emotional profile, the frontal, temporal and occipital lobes were mostly participating in emotional elicitation.

## 4. Discussion

EEG’s utility as a clinical tool for analyzing functional changes associated with different emotional states (anger, anxiety, disgust, happiness, sadness, surprise and neutral) across different brain areas (frontal, temporal, parietal and occipital scalp) is of considerable interest. In this regard, the research has established a novel conceptual connection between the SS, ES and TS profiles and the aforementioned emotional states across the brain regions. Conventional filters were employed to provide a preprocessing stage. A total of 14 channels across the various scalp regions were recorded while participants viewed seven brief emotional audio-visual video clips. The various domain features during this research, including spectral, entropy and temporal features, were computed so as to illustrate key EEG biomarkers relating to several emotional states. To provide more in-depth investigation, SS, ES and TS EEG emotional profiles were developed through the multivariate addition of spectral, entropy and temporal characteristics to spatial information. Overall, from the visual inspection of the spectral and temporal biomarkers, it was found that the frontal regions are particularly responsible for emotion detection while experiencing anger and anxiety in the upper-right quadrant of the valence-arousal circumplex model, whereas sadness and disgust appear in the lower-right left quadrant of the valence-arousal circumplex model. Surprise and happiness were situated in the upper-left quadrant of the valence-arousal circumplex model. The entropy biomarkers evidenced that the temporal regions were especially activated in the detection of emotion while experiencing anxiety and surprise, in the valence-arousal circumplex model’s upper-right and upper-left quadrants respectively. Table 8 presents the most highly correlated emotions with neutral for the SS, ES and TS profiles across the four brain regions. It was evidenced that the frontal, temporal, parietal and occipital lobes are primarily responsible for anger, anxiety and sadness elicitations. Emotions such as surprise are detectable in the frontal and parietal lobes whereas happiness and disgust may be elicited from the temporal and occipital lobes respectively. Accordingly, such findings imply that the combination of spectral, entropy and temporal feature sets could provide and convey more reliable biomarkers as a means of identifying SS, ES and TS profiles for anger, anxiety, disgust, happiness, sadness, surprise and neutral emotional states across the frontal, temporal, parietal and occipital scalp brain areas. The SS profile is significant in representing how anger emotions correspond to all brain lobes, while the ES profile is significant for representing anxiety emotions. Additionally, the TS profile is important for representing the sadness emotions.

Regarding the neuro-scientific perspective, all of the obtained results are consistent with the frontal, temporal, parietal and occipital brain lobes’ principal functions. The frontal lobe is deemed to be the emotional control center [83,84], while temporal lobes are linked to emotional perception [85]. Resultantly, to obtain greater insight into EEG emotional states, we incorporated several features from spectral, entropy and temporal aspects, enabling the identification of the most reliable EEG emotional biomarkers, as well as the development of the SS, ES and TS profiles as benchmarks for deeper inspection.

To sum up, emotions play a critical role in our day-to-day lives. Emotion investigation can gain a deeper understanding of human complex behavior. Emotions like happiness are considered as positive emotions that have been linked to a variety of outcomes including increased longevity and increased marital satisfaction [82]. Conversely, anger, anxiety and sadness are often thought of as negative emotions that have been linked to decreased life expectancy and may even have an impact on physical health [83,84]. Therefore, to capture and characterize people’s everyday emotional experiences, many recent scientific works validate the use of EEG as a diagnostic tool that is widely used in everyday life. So far, spectral, entropy and temporal biomarkers and SS, ES and TS EEG emotional profiles might be valuable physiological information that help in improving emotional investigation procedure.

## 5. Conclusions

In this study, EEG has been adopted for eliciting information in terms of waveform distribution over the scalp. The spectral, entropy and temporal biomarkers for emotion identification have been performed. These biomarkers were integrated to develop SS, ES and TS emotional profiles over the brain regions. The EEGs of 40 healthy volunteer students from the University of Vienna were recorded while they viewed seven brief emotional video clips. ANOVA has been conducted to identify the emotional biomarkers and Pearson’s correlations have been employed to determine the EEG emotion profiles. The results evidence that the combination of applied spectral, entropy and temporal sets of features may provide and convey reliable biomarkers for identifying SS, ES and TS profiles relating to different emotional states over the brain areas. EEG biomarkers and profiles enable more comprehensive insights into various human behavior effects as an intervention on the brain.

## Figures and Tables

**Table 1:** Sociodemographic data of the subjects with self-assessment questionnaire (SAQ) scores shown. (Age in years, SAQ mean ± standard deviation SD).

Demographic and Clinical Features		Subjects
**Number**		40
Age		22.475 ± 2.522
Female/Male		17F/23M
SAQ	Anger	4.052 ± 2.001
	Anxiety	1.844 ± 2.591
	Disgust	3.859 ± 2.843
	Happiness	2.204 ± 2.947
	Sadness	1.804 ± 2.365
	surprise	2.093 ± 2.438

**Table 2:** Emotions multiple comparison test using Bonferroni for the spectral biomarker.

(I) Emotion_Class	(J) Emotion_Class	Mean Difference (I-J)	${p-\mathrm{Value}\;}^{a}$
**Neutral**	Anger	0.659	0.123
	Anxiety	−0.096	1
	Disgust	−0.349	1
	Sadness	−0.891	0.004 *
	Surprise	0.114	1
	Happiness	−1.032	0.05 *
**Anger**	Anxiety	−0.755	0.033
	Disgust	−1.008	0.001
	Sadness	−1.55	0.05 *
	Surprise	−0.545	0.477
	Happiness	−1.691	0.05 *
**Anxiety**	Disgust	−0.253	1
	Sadness	−0.795	0.019 *
	Surprise	0.21	1
	Happiness	−0.936	0.002 *
**Disgust**	Sadness	−0.542	0.492
	Surprise	0.463	1
	Happiness	−0.683	0.09
**Sadness**	Surprise	1.005	0.001 *
	Happiness	−0.141	1
**Surprise**	Happiness	−1.146	0.05 *

* The mean difference is significant at the 0.05 level. ^a^ Adjustment for multiple comparisons: Bonferroni.

**Table 3:** Brain regions multiple comparison test using Bonferroni for the spectral biomarker.

(I) Brain Region	(J) Brain Region	Mean Difference (I-J)	${p-\mathrm{Value}\;}^{a}$
**Frontal**	Temporal	1.657	0.05 *
	Parietal	2.812	0.05 *
	Occipital	2.31	0.05 *
**Temporal**	Parietal	1.155	0.05 *
	Occipital	0.653	0.038
**Parietal**	Occipital	−0.502	0.215

* The mean difference is significant at the 0.05 level. ^a^ Adjustment for multiple comparisons: Bonferroni.

**Table 4:** Emotions multiple comparison test using Bonferroni for entropy biomarker.

Emotion	(J) Class_Emotion	Mean Difference (I-J)	${p-\mathrm{Value}\;}^{a}$
**Neutral**	Anger	−0.03	0.095
	Anxiety	−0.004	1
	Disgust	0.014	1
	Sadness	−0.016	1
	Surprise	−0.004	1
	Happiness	0.025	0.435
**Anger**	Anxiety	0.026	0.323
	Disgust	0.044	0.001 *
	Sadness	0.014	1
	Surprise	0.027	0.26
	happiness	0.055	0.05 *
**Anxiety**	Disgust	0.018	1
	Sadness	−0.012	1
	Surprise	0.001	1
	Happiness	0.029	0.134
**Disgust**	Sadness	−0.03	0.109
	Surprise	−0.017	1
	Happiness	0.011	1
**Sadness**	Surprise	0.012	1
	Happiness	0.041	0.003 *
**Surprise**	Happiness	0.028	0.169

* The mean difference is significant at the 0.05 level. ^a^ Adjustment for multiple comparisons: Bonferroni.

**Table 5:** Brain regions multiple comparison test using Bonferroni for entropy biomarker.

(I) Brain Region	(J) Brain Region	Mean Difference (I-J)	${p-\mathrm{Value}\;}^{a}$
**Frontal**	Temporal	−0.072	0.05 *
	Parietal	−0.062	0.05 *
	Occipital	−0.058	0.05 *
**Temporal**	Parietal	0.01	1
	Occipital	0.014	1
**Parietal**	Occipital	0.004	1

* The mean difference is significant at the 0.05 level. ^a^ Adjustment for multiple comparisons: Bonferroni.

**Table 6:** Emotions multiple comparison test using Bonferroni for the temporal biomarker.

(I) Class_Emotion	(J) Class_Emotion	Mean Difference (I-J)	${p-\mathrm{Value}\;}^{a}$
**Neutral**	Anger	0.031	1
	Anxiety	0.051	1
	Disgust	−0.034	1
	Sadness	0.009	1
	Surprise	0.062	1
	Happiness	0.024	1
**Anger**	Anxiety	0.02	1
	Disgust	−0.065	1
	Sadness	−0.022	1
	Surprise	0.031	1
	Happiness	−0.007	1
**Anxiety**	Disgust	−0.085	1
	Sadness	−0.042	1
	Surprise	0.011	1
	Happiness	−0.027	1
**Disgust**	Sadness	0.043	1
	Surprise	0.096	0.837
	Happiness	0.058	1
**Sadness**	Surprise	0.053	1
	Happiness	0.015	1
**Surprise**	happiness	−0.038	1

^a^ Adjustment for multiple comparisons: Bonferroni.

**Table 7:** Brain regions multiple comparison test using Bonferroni for the temporal biomarker.

(I) Brain Region	(J) Brain Region	Mean Difference (I-J)	${p-\mathrm{Value}\;}^{a}$
**Frontal**	Temporal	0.183	0.05 *
	Parietal	0.169	0.05 *
	Occipital	0.136	0.001 *
**Temporal**	Parietal	−0.014	1
	Occipital	−0.047	1
**Parietal**	Occipital	−0.034	1

* The mean difference is significant at the 0.05 level. ^a^ Adjustment for multiple comparisons: Bonferroni.

**Table 8:** The most correlated emotions with neutral for spectro−spatial (SS), entropy−spatial (ES) and temporo−spatial (TS) profiles over the frontal, temporal, parietal and occipital brain regions.

Profiles	Frontal	Temporal	Parietal	Occipital
SS	anger, anxiety, sadness, surprise	anger, anxiety, happiness	anger, sadness, surprise	anger, sadness, surprise
ES	anxiety, sadness	anxiety, sadness	anxiety	anxiety
TS	sadness	sadness	anger, sadness, surprise	disgust
